# Spatially structured heterogeneity shapes large-scale cortical dynamics in a model of the human cortex

**DOI:** 10.1073/pnas.2532072123

**Published:** 2026-07-08

**Authors:** Leonardo Dalla Porta, Jan Fousek, Alain Destexhe, Maria V. Sanchez-Vives

**Affiliations:** ^a^Institute of Biomedical Investigations August Pi i Sunyer, Systems Neuroscience, Barcelona 08036, Spain; ^b^Central European Institute of Technology, Masaryk University, Brno 65691, Czech Republic; ^c^ Department for Integrative and Computational Neuroscience, Paris-Saclay University, CNRS, Paris-Saclay Institute of Neuroscience, Saclay 91400, France; ^d^ Catalan Institution for Research and Advanced Studies, Barcelona 08010, Spain

**Keywords:** hierarchical heterogeneity, brain states, neuromodulation, muscarinic, whole-brain modeling

## Abstract

The human brain displays substantial spatial variability in molecular, anatomical, and physiological organization. Yet, how this heterogeneity shapes large-scale neuronal dynamics remains poorly understood. To address this question, we employed a biologically informed large-scale cortical model capable of generating distinct brain states, from awake-like to sleep-like dynamics. Our model was constrained by empirical human structural connectivity (SC) and regional cholinergic muscarinic receptor (CHRM) maps derived from transcriptomic data, together with complementary positron emission tomography (PET)-based receptor maps. These regional maps were implemented as modulators of adaptation-related excitability. We found that modulating excitability according to the spatial organization of CHRM maps significantly impacted large-scale cortical dynamics: It not only facilitated network synchronization but also enhanced information flow between cortical regions. Importantly, these effects were conserved across transcriptomic and PET-derived maps and could not be fully reproduced by multiple null models preserving generic forms of heterogeneity. Moreover, we addressed a particularly intricate dynamic regime characterized by the coexistence of localized sleep-like activity within otherwise awake-like states. We showed that the emergence of these sleep-like slow waves was a byproduct of both regional levels of neuronal adaptation and SC. In summary, our findings highlight the critical role of molecular and anatomical heterogeneity in shaping widespread cortical dynamics, suggesting broad avenues for linking microscale diversity to macroscale function.

## Significance

Biological heterogeneity is a hallmark of brain organization, spanning molecular to anatomical scales, yet its impact on large-scale dynamics has remained largely unexplored. Here, we integrate spatially structured regional heterogeneity derived from muscarinic receptor maps into a biophysically grounded large-scale cortical model. We show that this structured heterogeneity enhances network synchronization and information flow, supporting more flexible and coordinated brain states. Moreover, we show that such a form of heterogeneity contributes to the emergence of localized sleep-like slow waves within an otherwise awake-like regime. These findings demonstrate that biologically aligned intrinsic heterogeneity is an important organizing principle governing cortex-wide communication and state transitions.

From wakefulness to sleep, the brain exhibits a rich repertoire of spatiotemporal dynamics that underlie behavior and cognition (1–3). These dynamics arise from the interplay of factors across multiple scales, including structural connectivity (SC), cellular physiology, and neuromodulatory signaling, each influencing both normal cortical function and susceptibility to pathology (4–6). Yet, the extent to which regional heterogeneity modulates large-scale brain dynamics remains an open question in contemporary neuroscience (7, 8), particularly across distinct brain states. Neuromodulators and neurotransmitters are well known to shape the functional and dynamical properties of cortical circuits (9–12), but their distribution across the cortex is far from uniform (4, 13). Instead, these molecular signals form spatially distinct hubs along the cortical hierarchy (13, 14). Despite this, how such molecular and regional heterogeneity gives rise to global brain dynamics and how this relationship depends on brain state remains poorly understood.

Computational models of the human brain have attempted to address this question by simulating large-scale neural dynamics (8, 15). However, most of these models have focused on the structural and synaptic properties, largely due to the earlier lack of detailed, molecular-level data. Recent advances in neuroimaging and transcriptomic techniques have advanced the field by enabling the construction of more biologically realistic heterogeneous brain models. Computational frameworks incorporating these region-specific features, such as the T1w:T2w ratio or estimates of excitation–inhibition balance, have demonstrated improved performance in capturing empirical functional dynamics (16–19).

Despite recent progress, the influence of molecular heterogeneity on global brain dynamics remains underexplored. Acetylcholine (ACh), for example, plays a central role in modulating brain states by influencing spike-frequency adaptation and cortical excitability (10, 20, 21). However, the cortical ACh levels vary with brain state and exhibit spatial heterogeneity reflecting nonuniform cholinergic projections (22–25). Notably, reduced cholinergic input correlates with spatially localized slow wave activity during REM sleep (26), raising broader questions about the role of cholinergic heterogeneity in generating sleep-like activity under both physiological and pathological conditions, such as attentional lapses or following brain lesions (27–29).

To address these questions, we built a biologically informed large-scale model of the human cortical circuit. Rather than assuming homogeneous excitability across cortical regions, the model incorporates intrinsic heterogeneity through an adaptation-related excitability parameter constrained by transcriptomic and PET-derived muscarinic acetylcholine receptor (mAChR) maps of the two most abundant mAChRs in the human cortex, namely, M1 and M2 (13, 30). By simulating a continuum of brain states (from awake- to sleep-like), we found that spatially structured regional heterogeneity significantly influences large-scale cortical dynamics when compared to its homogeneous counterpart and multiple null models. Beyond facilitating network coordination through synchronization, biologically aligned heterogeneity enhanced interregional communication, supporting more complex and flexible activity patterns. Furthermore, we show that spatially localized slow waves emerge naturally from the interplay between regional variations in neuronal adaptation and the underlying SC. Together, our findings underscore the critical role of spatially structured heterogeneity and anatomical connectivity in shaping large-scale cortical dynamics and offer a framework for linking microscale diversity to macroscale brain function.

## Results

mAChRs are known to modulate cortical excitability and local circuit dynamics through multiple cellular and synaptic mechanisms (31, 32). Building on this, we asked how spatially structured regional heterogeneity in the transcriptomic maps of M1 and M2 receptors influences large-scale cortical activity, and in particular, how it impacts asynchronous and synchronous activity patterns, dynamics that closely resemble awake-like and sleep-like brain states, respectively. To this end, we incorporated these receptor maps into a biophysically grounded large-scale cortical model through an adaptation-related excitability parameter.

### Framework Overview.

**Fig. 1.**
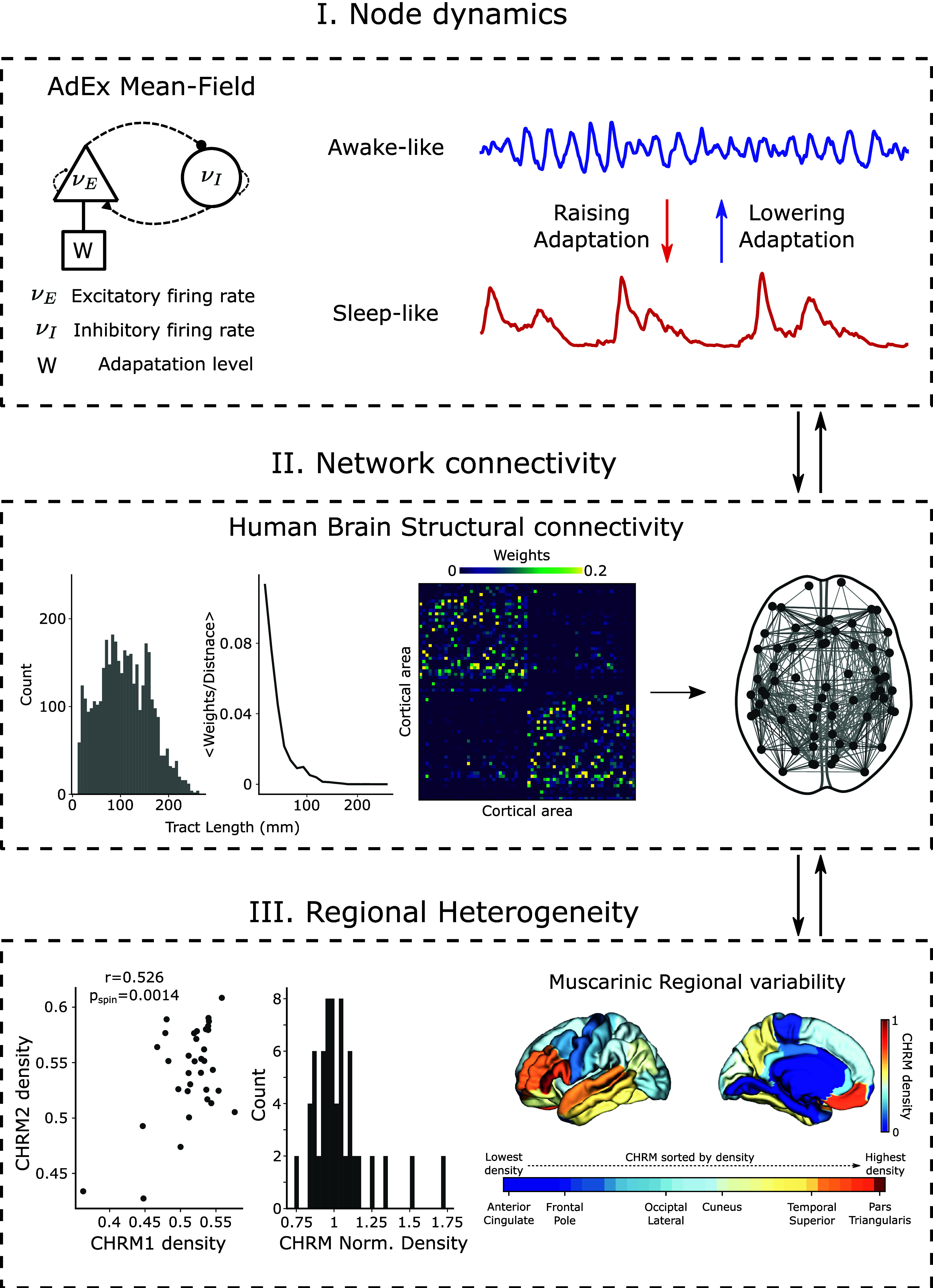
Overview of the large-scale cortical network model framework. *Top* panel: Each cortical region (node) was modeled using the Adaptive Exponential Mean-Field (AdEx-MF) model. In this model, excitatory (E) and inhibitory (I) neuronal populations are coupled together along with an adaptation variable (W). By varying the adaptation strength, the model can exhibit distinct dynamical regimes: low adaptation leads to asynchronous, awake-like activity, whereas high adaptation produces synchronous, sleep-like activity. *Middle* panel: Network connectivity was defined using empirical SC data derived from human diffusion MRI tractography. The brain was parcellated into 68 cortical regions according to the Desikan-Killiany atlas, and connection strength and transmission delays were estimated from diffusion tensor imaging (DTI) data. Interregional connectivity was set as excitatory. The preprocessed data were obtained from ref. 36. *Bottom* panel: Regional heterogeneity was introduced in the form of mAChR density. Gene expression levels of M1 (CHRM1) and M2 (CHRM2) receptor subtypes were highly correlated (r = 0.526, p_spin_ = 0.0014), and their normalized average (CHRM density) was used to modulate the local levels of excitability through the adaptation parameter (see *Materials and Methods* for details). Gene expression profiles were obtained from the Allen Human Brain Atlas (AHBA) and processed using the ABAGEN toolbox (13, 37).

Our large-scale modeling framework integrates three key components: local dynamics, network connectivity, and regional heterogeneity (Fig. 1). Each cortical region (node) was modeled using the adaptive exponential mean-field (AdEx-MF) model, which captures the interaction between excitatory ($\nu_{E}$) and inhibitory ($\nu_{I}$) neuronal populations, along with an adaptation variable (W) (33). *W* represents activity-dependent processes that modulate neuronal excitability, such as activity-dependent K^+^ currents, a known target of cholinergic modulation (10, 12, 31, 34). The dynamic balance between excitation, inhibition, and adaptation allows each node to exhibit either asynchronous (awake-like) or synchronous (sleep-like) dynamics, depending on the level of adaptation. Specifically, increasing neuronal adaptation drives the system toward a synchronous pattern of activity, while lower adaptation promotes irregular, desynchronized activity (Fig. 1, *Top* panel) (33, 35).

To define network connectivity, we used empirical SC data derived from human diffusion MRI tractography (Fig. 1, middle panel; see *Materials and Methods*) (36). The brain was parcellated according to the Desikan-Killiany atlas (68 cortical regions), and interregional connections were modeled as excitatory. Connection strengths (weights) and transmission delays were based on estimates of fiber density and tract length, respectively, obtained from DTI data (36).

Finally, regional heterogeneity was implemented by incorporating spatial variation in cortical muscarinic receptor density (Fig. 1, bottom panel). Specifically, we used the average expression levels of mAChR subtypes M1 (CHRM1) and M2 (CHRM2), the two most abundant muscarinic receptors in the human cortex (13, 30). Gene expression profiles were obtained from the AHBA and processed using the ABAGEN toolbox (13, 37, 38). These regional maps were used to modulate the adaptation parameter *W* in each node, thus linking neuromodulatory heterogeneity to local excitability (34). Although cholinergic signaling involves multiple pathways beyond the modulation of intrinsic excitability, it is well established that muscarinic receptor activation suppresses several potassium conductances underlying neuronal adaptation, thereby providing a physiological motivation for modeling regional muscarinic receptor expression as a reduced-order proxy of adaptation strength (12, 31, 32, 34). These adaptation-mediating potassium currents have also been shown to play a central role in the genesis of cortical slow oscillations, thereby linking muscarinic modulation of adaptation to the emergence of brain state transitions between sleep-like dynamics and wakefulness (39–41).

Altogether, this approach allowed us to construct a spatially heterogeneous virtual brain model in which transcriptomic muscarinic maps defined the primary axis of regional heterogeneity, while complementary analyses using independent PET-derived maps were used to assess robustness across molecular modalities.

### From Asynchronous to Synchronous Dynamics in a Large-Scale Cortical Network Model.

**Fig. 2.**
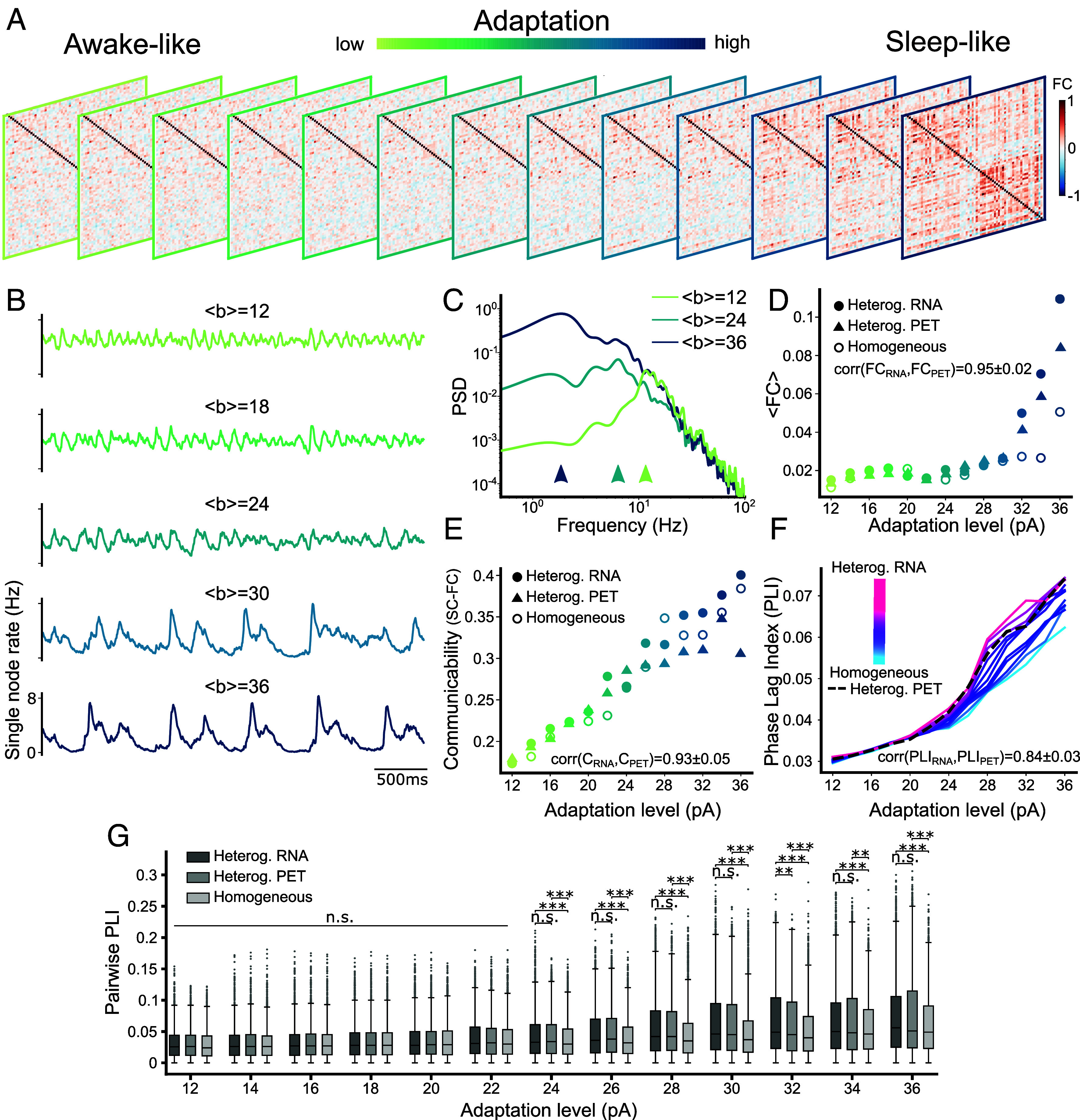
Heterogeneous large-scale cortical model from asynchronous to synchronous dynamics. (*A*) FC (Pearson correlation) matrices as a function of adaptation levels (color coded). (*B*) Representative time series of a single cortical region for different adaptation levels (b in pA units; <> represents the average over all cortical regions). (*C*) Power spectral density (PSD) for three representative adaptation levels illustrating awake-like (<b> = 12 pA), intermediate (<b> = 24 pA), and sleep-like (<b> = 36 pA) dynamics. Arrows indicate the peak frequency at ~11 Hz, ~6 Hz, and ~2 Hz, respectively. (*D*) Mean FC as a function of adaptation levels for transcriptomic-constrained heterogeneous (RNA; filled circles), PET-constrained heterogeneous (filled triangles), and homogeneous (empty circles) networks. (*E*) Same as (*D*) for SC-FC coupling [assessed using the weighted communicability measure (42)]. (*F*) Phase lag index (PLI) as a function of adaptation level for networks with different degrees of heterogeneity. Colored solid lines indicate RNA heterogeneous networks ranging from homogeneous to fully heterogeneous configurations, and black dashed-line indicates PET heterogeneous network. (*G*) Distribution of pairwise PLI values across cortical regions as a function of adaptation level for RNA-constrained heterogeneous (dark gray), PET-constrained heterogeneous (gray), and homogeneous (light gray) networks. Statistical significance was evaluated using an independent two-sample *t* test (***P < 0.001; **P < 0.01; *P < 0.05; n.s., not significant). See *SI Appendix*, Fig. S3 for FC comparisons between RNA- and PET-constrained networks.

Following our previously described framework, we next investigated the global cortical network dynamics as a function of the adaptation level (b; Fig. 2). At low adaptation levels (<b> = 12 pA; <> indicates average over all cortical regions), the system exhibited weak interregional correlations, as illustrated by the functional connectivity (FC) matrix, resembling the desynchronized activity typically associated with awake-like states (Fig. 2 *A*–*D*). Accordingly, temporal fluctuations in this regime were characterized by low-amplitude, high-frequency activity, which gave rise to irregular oscillations in the range of ~10 Hz, also accompanied by a suppression of the low-frequency content (Fig. 2 *B* and *C*). Consequently, this desynchronized state was dominated by irregular fluctuations, resulting in low correlation values across brain regions (Fig. 2*D*).

As adaptation increased (<b> = 24 pA), interregional correlations also increased and locally connected clusters began to emerge, giving rise to structured spatiotemporal patterns (Fig. 2*A*). In this intermediate regime, network firing rate decreased (from ~6 to ~4 Hz), and the dominant oscillatory frequency slowed (from ~10 to ~6 Hz), alongside a relative increase in low-frequency content (Fig. 2 *B* and *C*). Despite the emergence of localized synchrony, as indicated by the FC matrices, the global network remained weakly correlated (Fig. 2*D*; *Emergence of Localized Sleep-Like Slow Waves*). Importantly, these global network dynamics emerged through modulation of local dynamic properties as a function of spatially heterogeneous CHRM density ($\rho_{\mathrm{CHRM}}$). Specifically, regions with higher $\rho_{\mathrm{CHRM}}$ exhibited faster, lower-amplitude fluctuations, consistent with lower effective adaptation values, whereas regions with lower $\rho_{\mathrm{CHRM}}$ displayed progressively slower and more synchronized activity patterns across dynamical regimes, together with an enhancement in the delta-power band (*SI Appendix*, Fig. S1).

For higher adaptation levels (<b> = 36 pA), network dynamics were dominated by strongly correlated fluctuations across widespread cortical regions, a dynamic regime reminiscent of SWS and deep anesthesia (Fig. 2*A*) (43–45). In this state, the system exhibited quasi-periodic (~1 Hz) alternations between periods of high-amplitude (~8 Hz) sustained activity (Up states) and periods of network silence (Down states or off-periods), which propagated through the network as traveling waves (Fig. 2*B* and *SI Appendix*, Fig. S2) (21). As a result, low-frequency components dominated the system, leading to a globally synchronized network state (Fig. 2 *C* and *D*).

Another relevant feature distinguishing awake- and sleep-like states is structure–function coupling, i.e., the relationship between the underlying anatomical structure and the emergent functional patterns (46, 47). Our model reproduced this relationship accurately (Fig. 2*E*). Specifically, for awake-like states (desynchronized activity), the FC was only loosely constrained by the SC. Conversely, during sleep-like states (synchronized activity), the FC patterns were more constrained by the SC. This strengthened FC–SC coupling is thought to reflect a reduced repertoire of dynamic brain states, which is typically higher during wakefulness and significantly lower during sleep and unconscious states (46, 48).

Importantly, these dynamic features were conserved across both transcriptomic- and PET-derived muscarinic maps (Fig. 2 *D* and *E*). Transcriptomic CHRM1/2 maps and PET-derived M1 receptor maps exhibited significant spatial correspondence (*SI Appendix*, Fig. S3), and both implementations generated highly similar FC organization and structure–function coupling across dynamical regimes. Edgewise FC correlations between transcriptomic and PET implementations remained consistently high across awake-like, intermediate, and sleep-like regimes (Fig. 2*D*), indicating that the principal network-level signatures were robust across molecular modalities rather than specific to transcriptomic data alone.

### Regional Heterogeneity Facilitates Large-Scale Synchronization.

In the previous section, we demonstrated that our large-scale cortical network model, incorporating regional heterogeneity constrained by the spatial distribution of transcriptomic mAChR density, can reproduce key features of both awake-like and sleep-like brain dynamics (Fig. 2). However, a crucial question remains: How does the inclusion of spatially organized intrinsic regional heterogeneity impact global, widespread cortical dynamics? To address this, we compared our heterogeneous model to its homogeneous counterpart by keeping the adaptation level constant across brain regions, i.e., heterogeneity = 0% (<b> = b).

By comparing FC profiles across adaptation levels, we found that regional heterogeneity facilitated network synchronization; however, only at higher adaptation values (Fig. 2*D*; compare filled vs. empty circles). For adaptation levels above <b> = 30 pA, the mean FC (measured via Pearson correlation) was higher in the heterogeneous model, indicating that regional variability in adaptation enhances interregional coupling under synchronized states (Fig. 2*D*). Interestingly, although the heterogeneous network exhibited stronger overall synchronization, the structure–function coupling remained similar (Fig. 2*E*). That is, the relationship between FC and SC did not significantly differ between the heterogeneous and homogeneous models, suggesting that, despite higher synchronization, the dynamics remained constrained by the underlying anatomical structure. PET-constrained models exhibited highly similar FC–SC coupling profiles across adaptation levels (Fig. 2*E*).

In our model, synchronized regimes were characterized by Up and Down dynamics (Fig. 2*B*), which propagate through the network as traveling waves (*SI Appendix*, Fig. S2) (45). Thus, to further investigate the impact of regional heterogeneity on network synchronization, we computed the PLI (*Materials and Methods*). PLI is a metric designed to quantify phase-lagged synchronization while minimizing the influence of zero-lag correlations (49). Thus, PLI is particularly well-suited for detecting nontrivial phase relationships, such as those expected during traveling wave dynamics (45).

PLI analysis confirmed that regional heterogeneity indeed enhances network synchronization (Fig. 2*F*). Across adaptation levels, both transcriptomic and PET-constrained models exhibited progressively higher phase synchronization relative to the homogeneous counterpart. This difference became more pronounced at higher adaptation levels associated with stronger global synchronization. Moreover, synchronization increased progressively with the degree of imposed heterogeneity, indicating a continuous relationship between structured regional heterogeneity and large-scale cortical coordination (Fig. 2*F*).

To further characterize synchronization at the level of pairwise regional interactions, we quantified the distribution of pairwise PLI values across cortical regions (Fig. 2*G*). For low adaptation levels, synchronization remained weak and comparable across heterogeneous and homogeneous conditions. However, as adaptation increased, both transcriptomic- and PET-constrained models exhibited significantly higher pairwise synchronization relative to the homogeneous condition. Importantly, transcriptomic and PET implementations displayed similar synchronization profiles across adaptation regimes, indicating that the observed enhancement of large-scale coordination was robust across molecular modalities.

### Specificity of Regional Heterogeneity Effects.

To determine whether the observed enhancement of phase synchronization, measured by PLI (Fig. 2*F*), reflects the specific spatial organization of mAChRs rather than generic variability, we compared simulations using the biologically aligned receptor map against multiple null models that progressively remove structural information (*SI Appendix*, Figs. S4 and S5). Importantly, all null models could generate transitions between awake-like and sleep-like states, indicating that the mere presence of heterogeneity was sufficient to preserve broad state transitions. We therefore focused on whether biologically aligned heterogeneity selectively enhanced large-scale coordination beyond these generic effects.

To quantify these differences, we computed z-scores by standardizing the aligned-map PLI with respect to the corresponding null distributions. First, spatial shuffling of the receptor map reduced PLI values relative to the aligned configuration, indicating sensitivity to regional organization (Table 1). Second, replacing the map with random values drawn from a Gaussian distribution matched for mean and variance resulted in only weak differences in intermediate regimes (z ~ 1.5) and moderate differences in sleep-like regimes (z ~ 2; Table 2). This result indicates that generic heterogeneity already captures part of the observed dynamical behavior, while biologically structured heterogeneity provides an additional and selective enhancement of large-scale coordination. Finally, spin-rotated surrogate maps (50, 51) preserving spatial autocorrelation but disrupting anatomical alignment consistently reduced PLI across both intermediate (z ~ 1.8 to 3.3) and sleep-like regimes (z ~ 2 to 2.3) (Table 3).

**Table 1.** Control analysis using shuffled heterogeneity maps

Variables/States	Adaptation level (pA)	Heterogeneity factor	PLI aligned	PLI shuffled	ΔPLI	Standardized differences (z)	Empirical *P*-value
Intermediate	<24>	0.5	0.0364	0.0355	0.0009	0.7292	0.4752
Intermediate	<24>	1	0.04	0.0365	0.0035	1.8515	0.0396
Sleep-like	<36>	0.5	0.0707	0.0652	0.0055	2.8925	0.0099
Sleep-like	<36>	1	0.073	0.0654	0.0077	2.3588	0.0297

Comparison of PLI values obtained using the biologically aligned muscarinic receptor map and spatially shuffled surrogate maps. Results are shown for intermediate and sleep-like regimes at different heterogeneity levels. ΔPLI represents the difference between aligned and null mean PLI values, and z values denote standardized differences between the aligned map and the null distribution (100 surrogates per condition), computed as the difference from the null mean divided by the null SD. Higher z values indicate stronger phase-lagged synchronization for the aligned map compared to spatially randomized configurations. Empirical *P*-values were computed by comparing the observed aligned-map PLI against null distributions obtained from shuffled receptor maps (two-sided).

**Table 2.** Control analysis using Gaussian-distributed heterogeneity maps matched for mean and variance

Variables/States	Adaptation level (pA)	Heterogeneity factor	PLI aligned	PLI shuffled	ΔPLI	Standardized differences (z)	Empirical *P*-value
Intermediate	<24>	0.5	0.0364	0.0358	0.0006	0.4196	0.7419
Intermediate	<24>	1	0.04	0.0369	0.003	1.5179	0.1089
Sleep-like	<36>	0.5	0.0707	0.0651	0.0056	2.5046	0.009
Sleep-like	<36>	1	0.073	0.0650	0.008	1.9883	0.0697

Comparison of PLI values obtained using the biologically aligned muscarinic receptor map and random surrogate maps drawn from a Gaussian distribution matched to the empirical mean and variance. Results are shown for intermediate and sleep-like regimes at different heterogeneity levels. ΔPLI represents the difference between aligned and null mean PLI values, and z values denote standardized differences between the aligned map and the null distribution (100 surrogates per condition), computed as the difference from the null mean divided by the null SD. Higher z values indicate stronger phase-lagged synchronization for the aligned map compared to spatially randomized configurations. Empirical *P*-values were computed by comparing the observed aligned-map PLI against null distributions obtained from shuffled receptor maps (two-sided).

**Table 3.** Control analysis using spin-rotated (preserved spatial autocorrelation) surrogate maps

Variables/States	Adaptation level (pA)	Heterogeneity factor	PLI aligned	PLI shuffled	ΔPLI	Standardized differences (z)	Empirical *P*-value
Intermediate	<24>	0.5	0.0364	0.0344	0.002	1.8642	0.0792
Intermediate	<24>	1	0.04	0.0342	0.0057	3.3209	0.0099
Sleep-like	<36>	0.5	0.0707	0.0601	0.0106	2.1587	0.0891
Sleep-like	<36>	1	0.073	0.0586	0.0145	2.2731	0.0297

Comparison of PLI values obtained using the aligned muscarinic receptor map and spatially autocorrelation-preserving surrogate maps generated via spin rotation on the cortical surface. Results are shown for intermediate and sleep-like regimes at different heterogeneity levels. ΔPLI represents the difference between aligned and null mean PLI values, and z values denote standardized differences between the aligned map and the null distribution of spin-rotated maps (100 surrogates per condition), computed as the difference from the null mean divided by the null SD. Higher z values indicate stronger phase-lagged synchronization for the aligned map compared to spatially randomized configurations. Empirical *P*-values were computed by comparing the observed aligned-map PLI against null distributions obtained from shuffled receptor maps (two-sided).

Although several null conditions still generated qualitatively similar dynamical regimes, biologically aligned heterogeneity consistently produced stronger phase synchronization and interregional coordination, particularly at higher adaptation levels associated with globally synchronized activity. Together, these results indicate that generic regional heterogeneity is sufficient to preserve broad dynamical transitions, whereas biologically structured heterogeneity selectively enhances large-scale coordination within synchronized regimes.

### Information Flow Is Enhanced in Large-Scale Networks with Regional Heterogeneity.

**Fig. 3.**
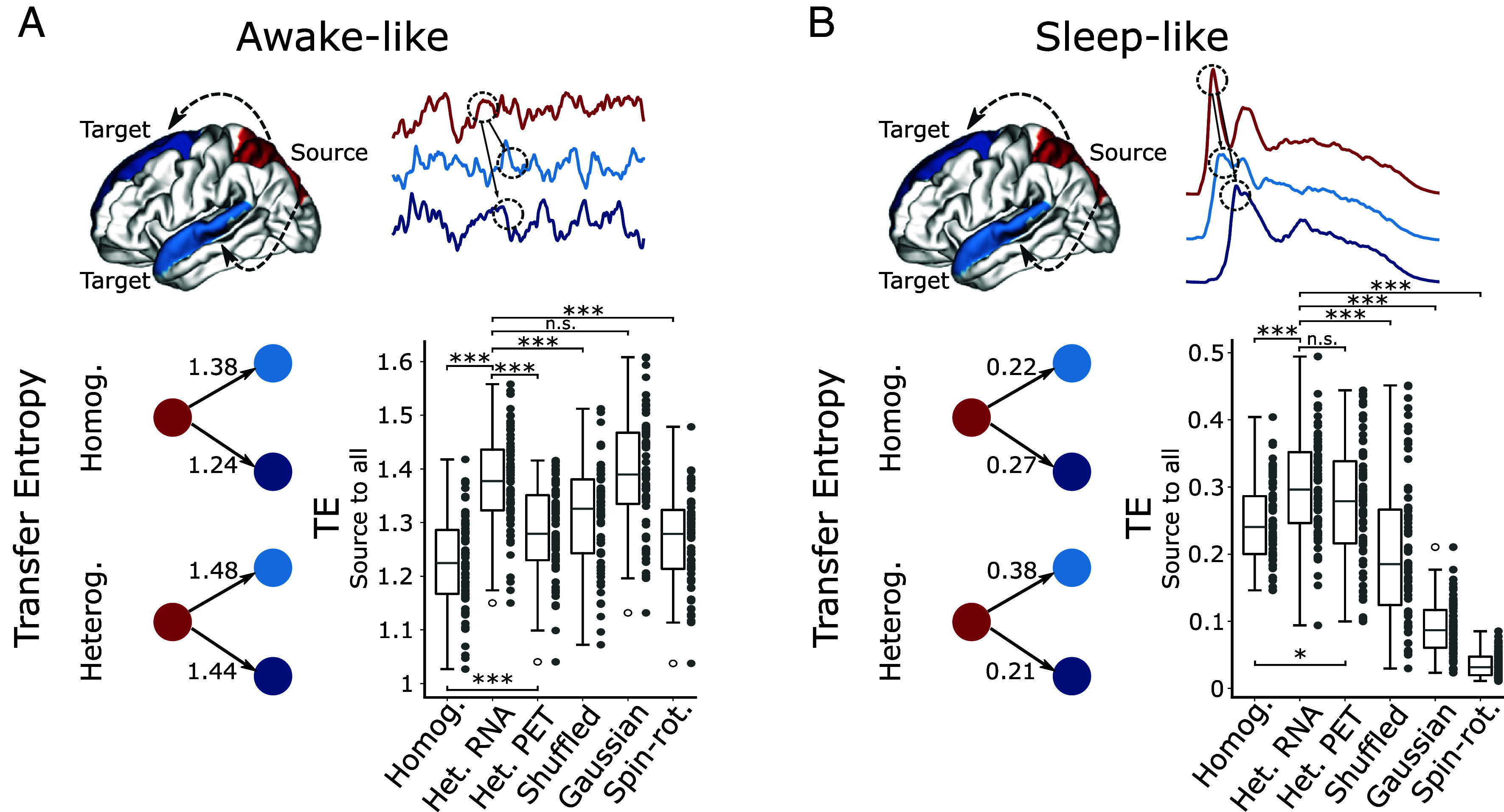
Information flow is enhanced in spatially heterogeneous large-scale cortical networks. (*A*) TE was computed from the stimulated area (source) to other cortical areas during the awake-like regime for transcriptomic-constrained (RNA) heterogeneous (<b> = 10 pA), PET-constrained heterogeneous (<b> = 10 pA), and homogeneous (b = 10 pA) networks, as well as for null models, namely shuffled, Gaussian-distributed and spin-rotated maps (see *Materials and Methods* for details). *Bottom Left* illustrates TE between two example region pairs, highlighted in the brain illustration above. *Bottom Right* shows the full TE profile from the source to all cortical regions. (*B*) Same as in (*A*), but for the sleep-like regime, comparing heterogeneous (<b> = 50 pA) and homogeneous (b = 50 pA) networks as well as null models. Statistical significance was evaluated using an independent two-sample *t* test (***P < 0.001; **P < 0.01; *P < 0.05; n.s., not significant).

A fundamental aspect of neuronal networks is their ability to receive and transmit information across the connectome, a process supported by both local and long-range connections (52–54). While previous studies have shown that information flow is enhanced in local spiking networks with heterogeneous neuronal properties, this effect has been less explored at the large-scale (7, 55–57). To address this, we assessed information flow across the connectome in both heterogeneous and homogeneous networks, as well as in different null models. To this end, we briefly stimulated a single cortical area under both awake-like and sleep-like dynamic regimes and computed the transfer entropy (TE) (58) between the stimulated region and all other areas (Fig. 3).

We found that, regardless of brain state, awake-like or sleep-like, TE was significantly higher in both transcriptomic- and PET-constrained heterogeneous networks compared to their homogeneous counterparts (Fig. 3). During the awake-like regime, when the network was more excitable and exhibited asynchronous dynamics, the mean TE (from source to all other areas) in heterogeneous networks was significantly higher than in homogeneous ones (1.38 ± 0.09 vs. 1.23 ± 0.08 and 1.28 ± 0.08 vs. 1.23 ± 0.08, for RNA and PET, respectively) and also significantly higher than in the null models, except for the Gaussian-distributed maps, for which no statistical significance was observed (Fig. 3*A*).

Similarly, in the sleep-like regime, characterized by Up and Down dynamics, TE remained higher in the heterogeneous condition when compared to the homogeneous case (0.30 ± 0.07 vs. 0.25 ± 0.06 and 0.28 ± 0.09 vs. 0.25 ± 0.06, for RNA and PET, respectively) and was also higher than in all null models tested (Fig. 3*B*), suggesting that regional variability supports more efficient information flow even under globally synchronized states. Moreover, TE values were also consistently higher in awake-like states compared to sleep-like states, in agreement with previous findings that Up and Down dynamics are associated with a breakdown in effective connectivity and a reduced capacity for information processing (59, 60). Together, these results suggest that biologically structured regional heterogeneity enhances information flow in large-scale cortical networks across different brain states.

### Emergence of Localized Sleep-Like Slow Waves.

**Fig. 4.**
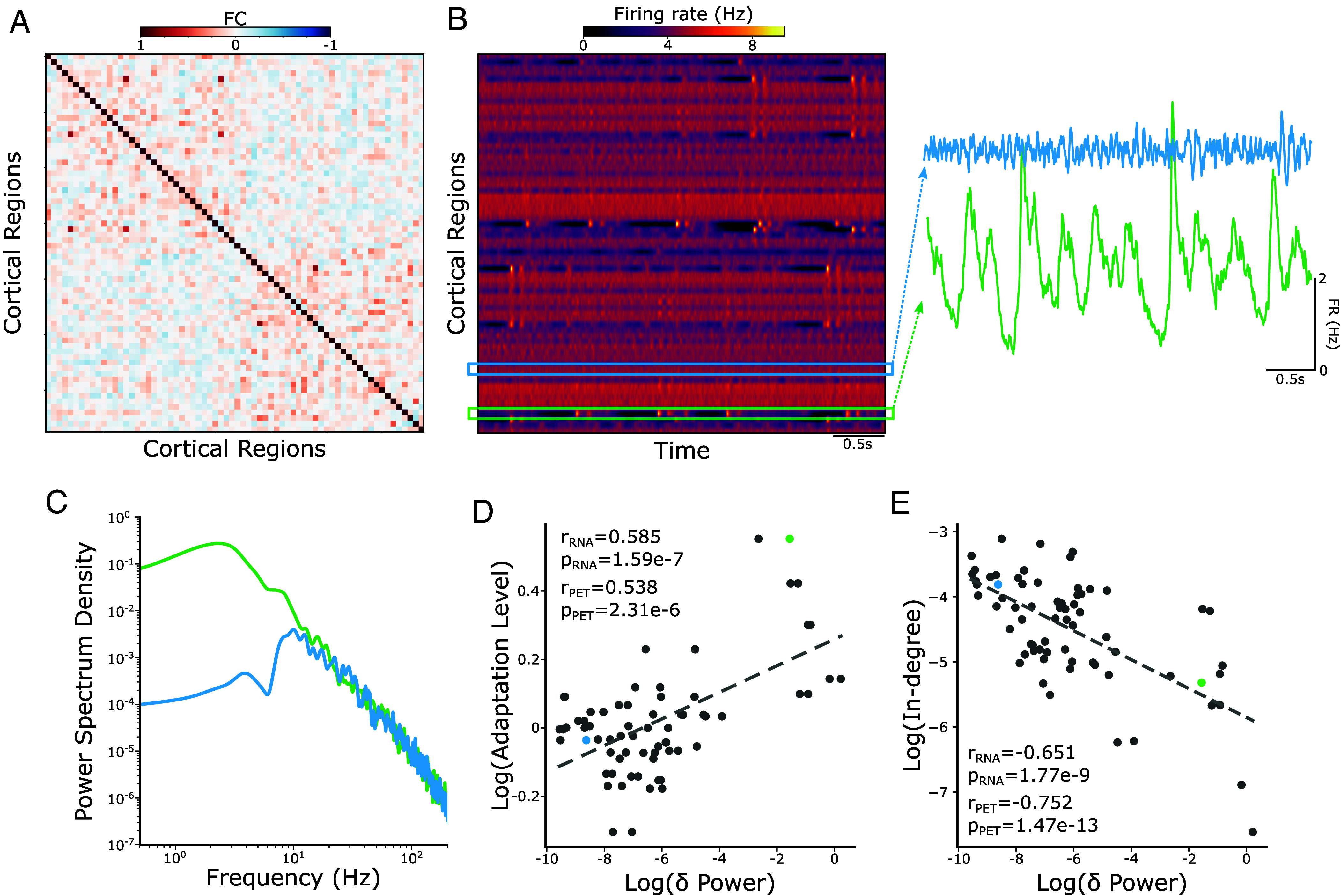
Emergence of localized sleep-like slow waves. (*A*) FC (Pearson correlation) matrix at an intermediate level of adaptation (<b> = 24 pA). (*B*) Raster plot of network firing rates. Blue and green rectangles highlight the coexistence of two distinct dynamics: awake-like and sleep-like, respectively. Corresponding time series are shown on the right. (*C*) Power spectrum density (PSD) for the regions highlighted in (*B*). (*D*) Correlation between adaptation level and delta power (mean PSD power in the 0 to 4 Hz band) across cortical regions. (*E*) Same as in (*D*) but showing the correlation between in-degree connectivity (average incoming input strength) and delta power. Pearson correlation coefficients and two-tailed p-values were used to assess statistical significance for both transcriptomic-constrained (RNA) heterogeneous and PET-constrained heterogeneous networks. See *SI Appendix*, Fig. S6 for correlation comparison against null models.

We now return to the intermediate level of adaptation (Fig. 2, <b> = 24 pA). As previously described, this regime was characterized by the emergence of locally synchronized clusters in the FC, while the global network remained only weakly correlated (Figs. 2 *A*–*D* and 4*A*). Here, we further examined the spatiotemporal features of this intermediate state and its underlying correlates (Fig. 4).

By plotting the firing rate of each brain region over time (Fig. 4*B*), we observed that a few brain regions exhibited sleep-like slow waves, i.e., Up and Down dynamics, while others maintained a persistent level of activity typical of awake-like states. These distinct temporal and spectral patterns, characterized by low-frequency, high-amplitude oscillations *vs* high-frequency, irregular fluctuations, coexisted across different regions within the same network (Fig. 4 *B* and *C*), revealing an intricate dynamic state for intermediate levels of adaptation.

However, what determines whether a region expresses sleep-like or awake-like dynamics in this intermediate regime? To investigate this question, we correlated the local adaptation levels with the delta power (mean power below 4 Hz; Fig. 4*D*). As expected, these two variables were highly positively correlated, indicating that regions with higher adaptation levels were more prone to shift into the sleep-like mode. Similar relationships were also observed in PET-constrained models (Fig. 4*D*). However, adaptation was not the only factor. The in-degree connectivity (average incoming input strength) also showed a strong negative correlation with delta power (Fig. 4*E*), suggesting that regions receiving fewer excitatory inputs were more prone to display sleep-like dynamics. Between adaptation levels and in-degree connectivity variables we observed a negative correlation, though statistically not significant (r = −0.23; *P* = 0.056). Together, these findings indicate that, in our model, localized sleep-like slow waves can emerge and coexist within an otherwise awake-like state, and that their spatial distribution is partially shaped by both regional adaptation levels and local connectivity properties. Moreover, our results are in line with recent empirical evidence that the level of regional cholinergic projections (from the basal forebrain) correlates inversely with the emergence of localized synchronous sleep-like activity (26).

Finally, we tested these relationships under the null hypothesis to dissociate the contributions of heterogeneity and SC. The positive correlation between regional adaptation and delta power was markedly attenuated under shuffled, Gaussian-distributed, and spin-rotated maps (*SI Appendix*, Fig. S6, top row), indicating that indeed the spatial organization of mAChR modulates the emergence of local sleep-like dynamics. In contrast, correlations between in-degree and delta power under null maps were consistently more negative than in the biologically aligned heterogeneous model and were also clustered near the homogeneous baseline (r_null_ ~ r_homo_; *SI Appendix*, Fig. S6, bottom row). Therefore, the spatially aligned mAChR map both strengthens the adaptation-delta relationship and reduces the extent to which delta power is trivially determined by the connectome, supporting a mechanism in which localized slow waves emerge from interactions between region-specific adaptation and network constraints. Together, these results suggest that disrupting mAChR alignment increases the dominance of SC in shaping slow wave propensity, whereas biologically aligned mAChR heterogeneity attenuates this dependence and shifts the balance toward adaptation-driven local dynamics.

## Discussion

Cortical dynamics are shaped by the connectome, as well as molecular and cellular composition, all of which vary across different brain regions (4, 13, 14, 52, 61). However, the extent to which this regional heterogeneity influences collective global brain dynamics has remained less explored, particularly across different brain states. In this study, we investigated the role of spatially structured regional heterogeneity using a biophysically grounded large-scale cortical network model constrained by empirical human connectivity and by regional muscarinic receptor maps derived from transcriptomic (13) and PET data (14). Regional heterogeneity was incorporated by modulating local node functional properties following the spatial organization of mAChRs, thereby creating a more detailed virtual brain landscape (Fig. 1). Our results show that aligned regional heterogeneity not only shapes spatiotemporal activity patterns by facilitating network synchronization but also enhances interregional communication. These findings underscore the significance of region-specific intrinsic heterogeneity in shaping the dynamics of large-scale networks.

Brain states span a multidimensional space, from highly correlated (e.g., slow wave sleep) to weakly correlated (e.g., awake, attentive states) states (2, 3). Transitions between these states are strongly influenced by neuromodulatory systems, which regulate neuronal excitability and network coordination (9, 10, 62, 63). Among these, ACh plays a central role in shaping brain state dynamics by reducing spike-frequency adaptation, thereby increasing cortical excitability and modulating network synchrony (21, 41, 64). ACh levels also fluctuate according to the brain state, being elevated during wakefulness and REM sleep, and decreasing during slow wave sleep (65, 66). Importantly, ACh release in the cortex, primarily stemming from the basal forebrain, is not spatially uniform, suggesting that neuromodulatory influence on neuronal dynamics may vary across brain regions (24–26, 67–69).

Here, we aimed to investigate how spatially structured regional heterogeneity shapes large-scale cortical dynamics (Fig. 1). To this end, we employed the AdEx-MF model constrained by human SC derived from tractography data (33, 36, 70). Although this model has previously been shown to reproduce both awake-like and sleep-like brain state dynamics (70, 71), those implementations assumed regional homogeneity. In the present study, we implemented regional heterogeneity through adaptation-related excitability parameters constrained by the spatial distribution of mAChRs M1 (CHRM1) and M2 (CHRM2) (13). These receptor subtypes are two of the most abundant muscarinic receptors in the human cortex and are key targets of cholinergic modulation (10, 30, 72). Importantly, our framework does not aim to capture the full range of cholinergic action (11, 66, 73); instead, we focus on a primary effect, namely modulation of cortical excitability (34), thereby creating a large-scale excitability gradient across the cortex. This approach is consistent with the level of abstraction commonly adopted in large-scale computational modeling (9, 20, 63, 71, 74–77). Accordingly, rather than providing a complete biophysical account of the cholinergic system, our model should be understood as a principled coarse-graining in which adaptation represents a low-dimensional effect of neuromodulator-induced changes in intrinsic currents, linking heterogeneous intrinsic cellular properties (a largely neglected factor in large-scale studies) to emergent large-scale dynamics in a tractable manner.

Our heterogeneous large-scale model reproduced key features of both awake- and sleep-like brain states (Fig. 2). During awake-like states, network dynamics were characterized by desynchronized patterns of activity and a suppression of low-frequency content. In contrast, sleep-like states were characterized by highly synchronous dynamics, dominated by propagating slow wave oscillations in the form of Up (active) and Down (silent) states (Fig. 2 and *SI Appendix*, Fig. S2). Additionally, the model also captured the brain state-dependent relationship between SC and FC (Fig. 2*E*). Specifically, during awake-like states, FC was only loosely constrained by the SC, whereas in sleep-like states, FC became more tightly aligned with SC. This strengthened FC–SC coupling has been observed in empirical human brain data and is thought to reflect a reduced repertoire of accessible dynamic states, a hallmark of unconscious states like deep sleep, compared to the broader dynamical flexibility present during wakefulness (46, 48, 78). Importantly, these dynamical signatures were consistently reproduced across both transcriptomic- and PET-constrained implementations.

Beyond reproducing key features of brain states, what is the impact of structured regional heterogeneity on large-scale cortical dynamics? Previous studies have shown that introducing spatially varying parameters such as local E:I balance, myelination gradients, or gene expression profiles significantly improves the fit of whole-brain models to empirical data (16–19). Heterogeneous models not only better replicate empirical brain activity but also support a richer dynamical repertoire, including more realistic regional timescales, ignition-like phenomena, and coherent spatiotemporal patterns (63, 77, 79, 80). Thus, regional heterogeneity is essential for bridging anatomical specialization and global functional organization in computational models of brain activity.

Here, we found that spatially structured regional heterogeneity facilitates large-scale synchronization when compared to its homogeneous counterpart (Fig. 2*D*). Using the mean FC as a proxy of network state dynamics, we found that, as a function of adaptation levels, heterogeneous networks tend to exhibit higher synchrony. Additionally, the PLI, which captures non-zero-lag correlations, confirmed that heterogeneous networks tend to synchronize more easily (Fig. 2*F*). Although this effect has been previously reported in systems of heterogeneous oscillators (81) or spiking neurons (57), our results extend this concept to a biologically realistic large-scale network model. Moreover, these effects were dependent on the empirical spatial organization of cholinergic muscarinic receptors, as neither randomized, Gaussian-distributed, nor spin-rotated maps reproduced the observed dynamics (Tables 1–3). Together, these findings suggest that biologically structured regional variability contributes to the emergence of coherent widespread cortical activity, an essential feature of the brain implicated in a diversity of physiological and cognitive processes (82–84).

Another fundamental effect of regional heterogeneity observed in our model was its influence on interregional communication. Previous work showed that during awake-like states, information transfer is facilitated, while during sleep-like states, most probably due to off-periods, information processing is hindered (59, 85–87). Our model was able to reproduce these empirical observations. Specifically, we showed that information transfer was greater during awake-like states compared to sleep-like states (Fig. 3). Moreover, both transcriptomic- and PET-constrained heterogeneous networks exhibited enhanced information flow when compared to homogeneous networks and across multiple null models (Fig. 3). This finding highlights the functional relevance of regional variability in supporting effective and flexible large-scale cortical communication, and aligns with prior work demonstrating that heterogeneity improves the brain’s capacity for information transmission and processing (88, 89).

Finally, we explored a special case: the coexistence of sleep-like slow waves within an otherwise awake brain. This complex phenomenon, where two distinct dynamic states coexist, has been previously reported under both physiological and pathological conditions. For example, in sleep-deprived awake rats, local slow waves have been observed within the awake brain and linked to impaired task performance (90). In humans, similar localized slow waves have also been observed during wakefulness, associated with attentional lapses under physiological conditions (27). Moreover, in both animals and humans, local slow waves have also been observed during physiological REM sleep (26, 29, 91–93). Such localized slow waves have also been reported during pathological conditions, including regions surrounding brain lesions and epileptogenic zones (28, 94–96). Thus, understanding the correlates of these local slow waves is crucial for elucidating both physiological and pathological brain processes.

Our model reproduced this phenomenon, in which localized sleep-like activity coexists within an overall awake-like brain state (Fig. 4). These localized slow waves were partially explained by regional heterogeneity in adaptation levels, consistent with a recent animal study reporting the occurrence of local slow waves during REM sleep associated with spatial variations in cholinergic innervation (26). Similar relationships were also observed in PET-constrained models. However, adaptation alone was not sufficient to fully explain the emergence of these localized slow waves. SC played a critical role, as the in-degree level of connectivity also modulates the regional level of excitability. This interplay is coherent with theoretical studies, where the level of adaptation and excitability are two key components of slow waves’ emergence and maintenance (97–100). Together, these findings suggest that localized slow wave activity arises from a complex interplay between molecular and structural features of the brain. Although we did not simulate pathological conditions, our framework could be extended to investigate how brain lesions and alterations in neurotransmitter levels might influence the generation of local slow waves after brain lesions (28, 94, 101–103), thus offering translational potential for developing personalized models of brain function and dysfunction.

In summary, our computational model highlights the impact of spatially structured regional heterogeneity on large-scale cortical dynamics. By incorporating molecular-level variability into a biophysically grounded whole-brain framework, our results suggest that biologically structured heterogeneity selectively enhances synchronization, information flow, and the emergence of localized sleep-like dynamics across distinct brain states. More broadly, these findings support a mechanistic link between regional molecular organization and large-scale cortical function (4, 104). Future work extending this framework to additional neuromodulatory systems, time-dependent neuromodulatory dynamics (105), or pathological conditions (103) may help clarify how regional heterogeneity contributes to physiological and pathological brain activity, including the emergence of localized sleep-like states following brain lesions or during disorders of consciousness. Nonetheless, despite recent advances in computational models of neuromodulation, fully integrating the multiscale and state-dependent aspects of neuromodulatory signaling into unified whole-brain frameworks remains an open challenge (8).

### Limitations of the Study.

Several limitations should be considered when interpreting the present findings. First, our implementation of muscarinic cholinergic modulation was intentionally simplified and focused specifically on adaptation-related changes in intrinsic excitability (31, 34). Although muscarinic receptors influence multiple cellular and synaptic processes (11, 12, 32, 67, 73, 106), our framework modeled only one dominant mechanism associated with cortical state regulation, namely the modulation of adaptation-mediating potassium currents that regulate neuronal excitability (32). Accordingly, the present model should be interpreted as a coarse-grained approximation of muscarinic cholinergic influences on cortical dynamics rather than a comprehensive physiological account of neuromodulatory signaling. Additionally, the present framework does not distinguish between the differential physiological roles of muscarinic receptor subtypes M1 and M2. Instead, transcriptomic CHRM1 and CHRM2 expression levels were combined into a single reduced-order parameterization of adaptation-related excitability, whereas the PET analyses relied exclusively on M1 receptor density. Consequently, receptor-specific signaling pathways and functional interactions associated with distinct muscarinic receptor subtypes are not explicitly represented in the current model (32, 107).

Second, although biologically aligned heterogeneity consistently enhanced synchronization and information flow relative to null conditions, several surrogate models still reproduced broad dynamical transitions between awake- and sleep-like regimes (*SI Appendix*, Figs. S4 and S5). This suggests that large-scale cortical dynamics do not depend exclusively on the precise molecular organization of heterogeneity, but rather emerge from interactions between intrinsic regional variability, adaptation dynamics, and the structural connectome. In this framework, regional heterogeneity appears to act primarily as a modulatory factor shaping large-scale coordination rather than as the sole determinant of network state transitions. Furthermore, because our model focused specifically on adaptation-related muscarinic effects, additional components of cholinergic signaling not captured here may also contribute to the organization of large-scale cortical dynamics.

Finally, transcriptomic and PET-derived receptor maps represent static population-level estimates and therefore do not capture the temporal fluctuations in neuromodulatory signaling that occur across behavioral and cognitive states (105). Future work incorporating time-varying neuromodulatory dynamics, additional neurotransmitter systems, or individualized connectomes may help clarify how molecular heterogeneity interacts with anatomical connectivity to shape physiological and pathological brain activity.

## Materials and Methods

### Mean-Field Model of Cortical Dynamics.

The dynamics of each node were simulated according to a mean-field (MF) description of networks of Adaptive Exponential Integrate and Fire neurons (AdEx), as previously described (33, 70, 71). Briefly, this model captures the interaction among excitatory (E) and inhibitory (I) neuronal populations. Also, excitatory populations are equipped with firing rate adaptation (W) dynamics. The dynamics equations read:

$$\begin{array}{c} T\frac{\partial \nu_{\mu }}{\partial t}=\left(F_{\mu }-\nu_{\mu }\right)+\frac{1}{2}c_{\lambda \eta }\frac{\partial^{2}F_{\mu }}{\partial \lambda \partial \eta },T\frac{\partial c_{\lambda \eta }}{\partial t}=\frac{F_{\lambda }(T^{-1}-F_{\eta })}{N_{\lambda }}+(F_{\lambda }-\nu_{\lambda })(F_{\eta }-\nu_{\eta })\\ +\frac{\partial F_{\lambda }}{\partial \nu_{\mu }}c_{\eta \mu }+\frac{\partial F_{\eta }}{\partial \nu_{\mu }}c_{\lambda \mu }-2c_{\lambda \eta },\frac{\partial W_{\mu }}{\partial t}=-\frac{W_{\mu }}{\tau_{W}}+b_{\mu }\nu_{\mu }\\ +\frac{a_{\mu }[\mu_{V}(\eta_{E},\eta_{I},W_{\mu })-E_{L\mu }]}{\tau_{W}}, \end{array}$$

where $\nu_{\mu }$ is the mean firing rate of the population μ={E,I}. $C_{\lambda \eta }$ is the covariance between population λ and η, $W_{\mu }$ is the mean adaptation, $b_{\mu }$ is the adaptation level (strength), and $a_{\mu }$ is the subthreshold adaptation, and T is the MF characteristic time constant. $F_{\mu }=F_{\mu }\left(\nu_{E},\nu_{I},W_{\mu }\right)$ is the transfer function (TF), which characterizes the dependence of the output firing rate on the excitatory ($\nu_{E}$) and inhibitory ($\nu_{I}$) inputs. According to ref. 108, it can be written as a function of its mean subthreshold membrane voltage $\mu_{V}$, its SD $\sigma_{V}$, and its correlation time decay $\tau_{V}$, as:

$$F=\nu_{\mathrm{out}}=\frac{1}{2\tau_{V}}\cdot Erfc\left(\frac{V_{\mathrm{thr}}^{\mathrm{eff}}-\mu_{V}}{\sqrt{2}\sigma_{V}}\right),$$

where ($\mu_{V}$, $\sigma_{V},\tau_{V}$) are obtained by solving a set of equations, as described in refs. 33 and 108. $V_{\mathrm{thr}}^{\mathrm{eff}}$ is a phenomenological spike threshold voltage taken as a second-order polynomial:

$$\begin{array}{c} V_{\mathrm{thr}}^{\mathrm{eff}}(\mu_{V},\sigma_{V},\tau_{V}^{N})=P_{0}+\sum_{x\in \{\mu_{V},\sigma_{V},\tau_{V}^{N}\}}P_{x}\left(\frac{x-x_{0}}{\partial x_{0}}\right)\\ +\sum_{x\in \{\mu_{V},\sigma_{V},\tau_{V}^{N}{\}}^{2}}P_{\mathrm{xy}}\left(\frac{x-x_{0}}{\partial x_{0}}\right)\left(\frac{y-y_{0}}{\partial y_{0}}\right), \end{array}$$

with $\tau_{V}^{N}=\frac{\tau_{V}g_{L}}{C_{m}}$, where $g_{L}$ is the leakage conductance and $C_{m}$ the membrane capacitance.

The constant values are defined as in ref. 71: $\mu_{V}=-60\;\mathrm{mV}$, $\sigma_{V}=0.004\;\mathrm{mV}$, $\tau_{V}^{N}=0.5$, $\delta \mu_{V}=0.001\;\mathrm{mV}$, $\delta \sigma_{V}=0.006\;\mathrm{mV}$, and $\sigma_{V}^{N}=1$. Accordingly, the fitted polynomials $P^{E,I}$, for *E* and *I* types of neurons are $P_{0}=-0.0498,-0.0514$, $P_{\mu V}=0.00506,0.004$, $P_{\sigma V}=-0.025,-0.0083$, $P_{\tau V}=0.0014,0.0002$, $P_{\mu^{2}V}=-0.00041,-0.0005$, $P_{\sigma^{2}V}=0.0105,0.0014$, $P_{\tau^{2}V}=-0.036,$-0.0146, $P_{\mu V\sigma V}=0.0074,0.0045$, $P_{\mu V\tau V}=0.0012,0.0028$, $P_{\sigma V\tau V}=$-0.0407,-0.0153, for (*E*, *I*), respectively. $\mu_{V}$ is a function that represents the average membrane potential of a given population, described by:

$$\mu_{V}=\frac{\mu_{G,E}E_{e}+\mu_{G,I}E_{i}+g_{L}E_{L}-W}{\mu_{G,E}+\mu_{G,I}+g_{L}}.$$

$\mu_{G,E}=\nu_{E}K_{E}u_{E}Q_{E}$, and the same applies to the *I* population. *Q* is the conductance weight, u is the synaptic time decay, and K=Np is a constant defined by the number of neurons ($N=10^{4}$) and the probability of connection (P=0.05). All parameters were obtained from (71) and were set to T=20 ms, $E_{L,\{E,I\}}=(-64,-65)\;\mathrm{mV}$, $N_{\left\{E,I\right\}}=\left(8000,2000\right)$, P=5%, $b_{I}=0\;\mathrm{pA}$, $a_{\left\{E,I\right\}}=(0,0)\;\mathrm{pA}$, $\tau_{W}=500\;\mathrm{ms}$, $g_{L}=10\;\mathrm{nS}$, $C_{m}=200\;\mathrm{pF}$, $Q_{\left\{E,I\right\}}=(1.5,5)\;\mathrm{nS}$, $\nu^{\mathrm{ext}}=0.315\;\mathrm{Hz}$, $K_{\left\{E.I\right\}}=\left(400,0\right)$, $E_{e,i}=\left(0,-80\right)$. $b_{E}$ sets the adaptation level of the excitatory populations and was used as a proxy of spatial cholinergic heterogeneity; see *Cholinergic Muscarinic Regional Heterogeneity*.

The previous equations describe the population dynamics of a single cortical region composed of excitatory and inhibitory populations. To describe large-scale networks of interconnected brain regions, each represented by the MF model, we can extend the TF to incorporate both interregional interaction and external noise. Thus, the TF can be rewritten as simply as:

$$F_{\mu }=F_{\mu }(\nu_{E}+\nu_{E}^{\mathrm{IN}},\nu_{I},W_{\mu }),$$

with,

$$\nu_{\mu }^{\mathrm{IN}}(k,t)=wOU_{k}(t)+G\sum_{j}C_{\mathrm{kj}}\nu_{E}(j,t-D_{k,j}).$$

*K* represents the node index, G=0.3 is the global coupling factor that scales all the connection weights, $C_{\mathrm{kj}}$ is the connectivity matrix strength between *j* and *k*, $D_{k,j}$ is the matrix delay of axonal propagation, computed as $||j-k||/\nu_{c}$, where ||j-k|| is the distance between nodes *j* and *k* and $v_{c}=5\;\mathrm{mm}/\mathrm{ms}$ is the propagation speed, w=1e-4 pA is the noise scaling factor, and $OU_{k}\left(t\right)$ is the noise defined by an Ornstein–Uhlenbeck process with $\tau_{\mathrm{OU}}=5\;\mathrm{ms}$.

### SC.

The SC was derived from human tractography data from the Berlin empirical data processing pipeline (36). A cortical parcellation of 68 brain regions was used, according to the Desikan-Killiany atlas. Connection strengths (weights) and transmission delays were based on estimates of fiber density and tract length, respectively, obtained from DTI data. The preprocessed data were obtained from ref. 36 and were the same used in a previous study (70).

### Cholinergic Muscarinic Regional Heterogeneity.

Regional heterogeneity was implemented by incorporating spatial variation in cortical cholinergic receptor density to modulate the level of excitability through neuronal adaptation in the AdEx-MF model. Gene expression data were obtained from the AHBA and were processed according to the ABAGEN toolbox using robust sigmoid normalization and RNA-sequencing-based probe selection, following established protocols for minimizing interdonor variability and preserving spatial fidelity (13, 17, 37, 38). Based on our knowledge of the role of cholinergic modulation in cortical circuits (31), we focused on muscarinic receptor genes. Specifically, mAChR subtypes M1 (CHRM1) and M2 (CHRM2), the two most abundant muscarinic receptors in the human cortex (30, 72). Because AHBA data contain more samples from the left hemisphere, we extracted expression values from the left cortex and mirrored them to the right hemisphere, consistent with previous modeling approaches (17).

In our implementation, we used transcriptomic data from the cholinergic muscarinic receptors CHRM1 and CHRM2. As their density was significantly correlated across regions (Fig. 1, *Bottom* panel; r = 0.526, *P*_spin_ = 0.0014), we worked with the normalized arithmetic mean, which we refer to simple as CHRM density. To ensure equivalence with a homogeneous network, we centered this distribution around its median, thus centering it around one (1.03 ± 0.20, mean and SD given). This normalized map was then inverted prior to implementation, such that regions with higher receptor density were assigned to lower effective adaptation values. The resulting CHRM density vector, containing 68 values (one per cortical region), was then used to modulate the adaptation parameter $b_{E}$ as:

$$b_{E}=C+b(1+h_{f}(\rho_{\mathrm{CHRM}}-1)).$$

C=10 pA is the baseline adaptation level, b is a free variable that represents the adaptation. $\rho_{\mathrm{CHRM}}$ represents the CHRM density, and $h_{f}$ is the heterogeneity factor, i.e., if $h_{f}=1$ the network is fully heterogeneous, while $h_{f}=0$ represents a fully homogeneous network.

PET data consisted of a muscarinic M1 receptor map (109) obtained from the *neuromaps* (14, 110) repository. PET values were standardized to zero mean and unit variance, transformed using a logistic sigmoid function, and subsequently rescaled as done for the gene expression data. Finally, PET values were centered around their median, analogous to the centering applied to gene expression data.

### Computational Simulations.

The model was run using The Virtual Brain (TVB) platform, where the AdEx-MF model is already implemented (111). Numerical integration was performed using the stochastic Heun’s method with a time step of 1 ms. For spontaneous activity simulations, each run lasted 22 s, with the first 2 s discarded to eliminate transients. For stimulation protocols, a brief excitatory input was applied to the firing rate of the excitatory population in a single region, the superior parietal lobule. The stimulus had a duration of 30 ms and an amplitude of 3 Hz. Each stimulation trial (50 trials) consisted of a new simulation lasting 6.03 s, with the first 2 s again discarded. The stimulus was applied at 4 s, allowing for a 2 s prestimulus and 2 s poststimulus window for analysis.

### Data Analysis.

FC and structure–function coupling (SC–FC) were estimated using the Pearson correlation, and the weighted communicability (42, 47), respectively. PSD was computed using Welch’s method (nfft = 10 and nperseg = 1). PLI was computed as described in ref. 49: $PLI=\left|<sign[\Delta \phi (t)]>\right|$ where Δϕ denotes the instantaneous phase difference between two signals. The instantaneous phase was estimated by the Hilbert transform. PLI measures the phase synchronization among signals while minimizing the influence of zero-lag correlations. PLI values range from 0 and 1, where 0 indicates no consistent phase lag, and 1 reflects perfect phase locking with a consistent nonzero phase difference. TE between two signals X(t) and Y(t), $TE_{X\to Y}$ was defined as:

$${\mathrm{TE}}_{X\to Y}=H(Y_{t}|Y_{t-1:t-\tau })-H(Y_{t}|Y_{t-1:t-\tau },X_{t-1:t-\tau }),$$

where H() denotes the entropy and τ=5 ms is the time delay. In short, $TE_{X\to Y}$ measures the reduction in uncertainty about the future of Y(t) given its own past and the history of a second variable X(t) (58, 112). We used the TE implementation available at https://github.com/notsebastiano/transfer_entropy. TE was computed from the source area to all other areas in a window of 400 ms after the stimulus (see above).

### Statistical Analysis and Null Models.

To assess the specificity of the effects of muscarinic heterogeneity maps, we compared simulations using the biologically aligned receptor map against three null models that progressively remove structural information. In the shuffled null model, receptor values were randomly permuted across cortical regions, preserving the empirical distribution while disrupting anatomical alignment. In the Gaussian null model, regional values were drawn from a normal distribution matched to the empirical mean and SD, testing whether heterogeneity magnitude alone could reproduce the observed effects. In the spatial null model, regional values were reassigned using spin rotations on the cortical surface as implemented in the ENIGMA toolbox (113), preserving spatial autocorrelation while disrupting regional correspondence. For each null model, 100 independent realizations were generated, and the full network model was simulated for each realization under identical parameters.

Correlations were quantified using Pearson’s correlation coefficient, with statistical significance assessed using two-tailed *P*-values. For spatial map comparisons (Fig. 1 and *SI Appendix*, Fig. S1), significance was evaluated using spatial autocorrelation-preserving permutation tests (spin tests; *P*_spin_) as implemented in the ENIGMA toolbox (50, 113, 114). For analysis reported in Table 1–3, observed values were compared against a null distribution generated from null models. Statistical significance was assessed using permutation-based methods: z values were obtained by standardizing observed statistics relative to the null distributions, and empirical two-sided *P*-values were computed as the proportion of surrogate realizations at least as extreme as the observed values. For the results presented in Fig. 3, statistical significance was evaluated using an independent two-sample *t* test. For analysis presented in *SI Appendix*, Fig. S3, permutation-based *P*-values were calculated by comparing observed correlation coefficients to distributions generated under the corresponding null models.

## Supplementary Material

Appendix 01 (PDF)

## Data Availability

The code needed to replicate the main findings of this study has been deposited in Heterogeneous Brain Model (https://github.com/ldallap/HeterogeneousBrainModel) (115). All other data are included in the manuscript and/or *SI Appendix*.
