# Supplementary material for: Spatially structured heterogeneity shapes large-scale cortical dynamics in a model of the human cortex
Source: Proc Natl Acad Sci U S A. 2026 Jul 8;123(28):e2532072123. doi: 10.1073/pnas.2532072123 (PMC13367858; doi:10.1073/pnas.2532072123)
Supplement: Supplementary file 1 — Appendix 01 (PDF) [file pnas.2532072123.sapp.pdf]

PNAS

Supporting Information for
Spatially structured heterogeneity shapes large-scale cortical dynamics in a model of the human cortex

Leonardo Dalla Porta, Jan Fousek, Alain Destexhe, Maria V. Sanchez-Vives

Leonardo Dalla Porta
Email: dallaporta@recerca.clinic.cat

This PDF file includes:

Figures S1 to S6

Figures

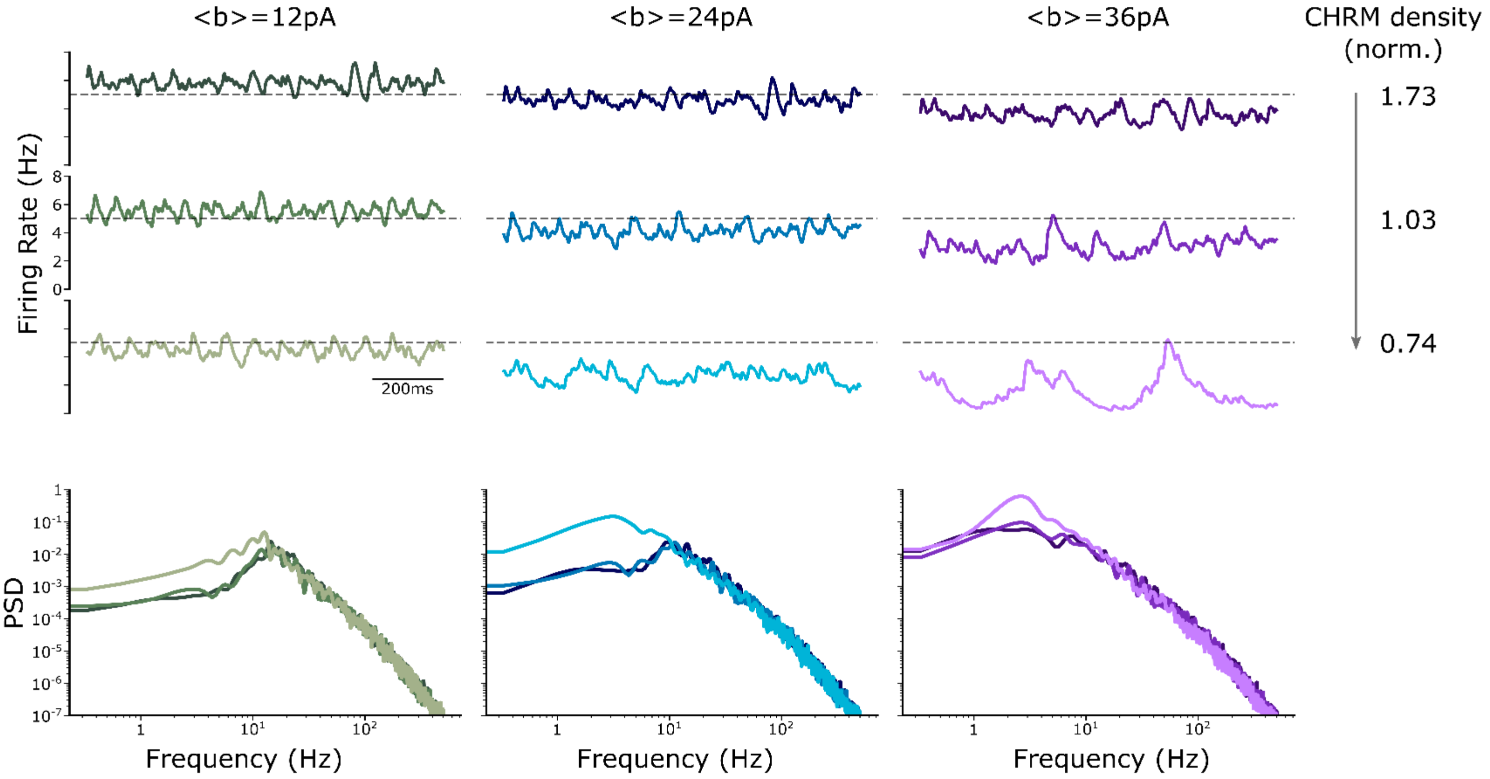

Fig. S1. Regional heterogeneity modulates local firing rate dynamics and spectral profiles. Representative firing rate time series (top) and corresponding power spectral density (PSD; bottom) for cortical regions with different CHRM-constrained adaptation levels across awake-like (<b>=12pA), intermediate (<b>=24pA), and sleep-like (<b>=36pA) dynamical regimes. Darker traces correspond to regions with higher normalized CHRM density values, whereas lighter traces indicate lower CHRM density. Regions with lower effective adaptation exhibited faster, lower-amplitude fluctuations, while regions with higher effective adaptation displayed progressively slower and more synchronized dynamics accompanied by enhanced low-frequency power.

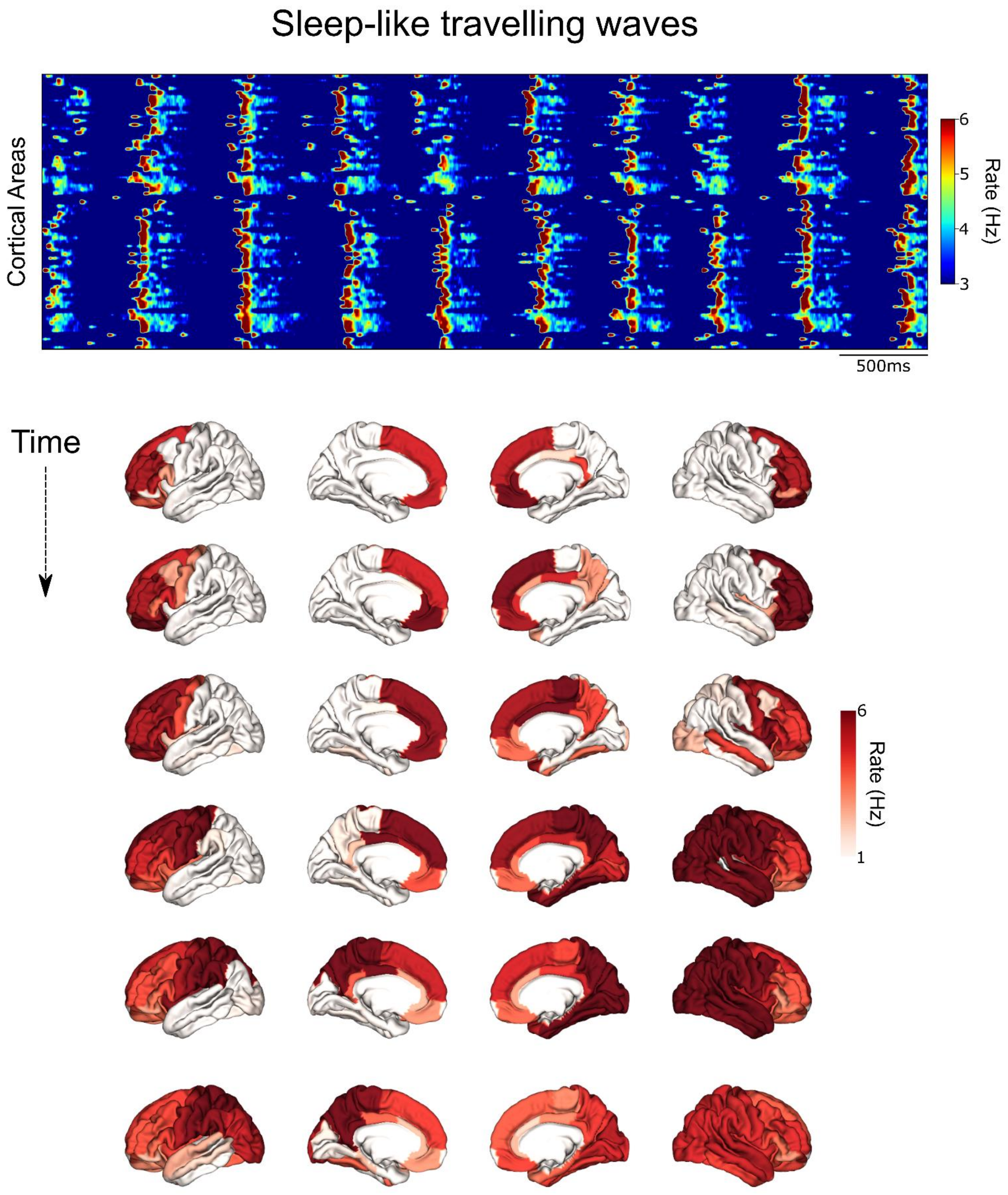

Fig. S2. Cortical travelling slow waves. Top, Raster plot of cortical areas illustrating Up (high firing rate) and Down (low firing rate) dynamics and wave propagation. Bottom, Illustration of a travelling wave during one Up state. Each row is separated by 20ms.

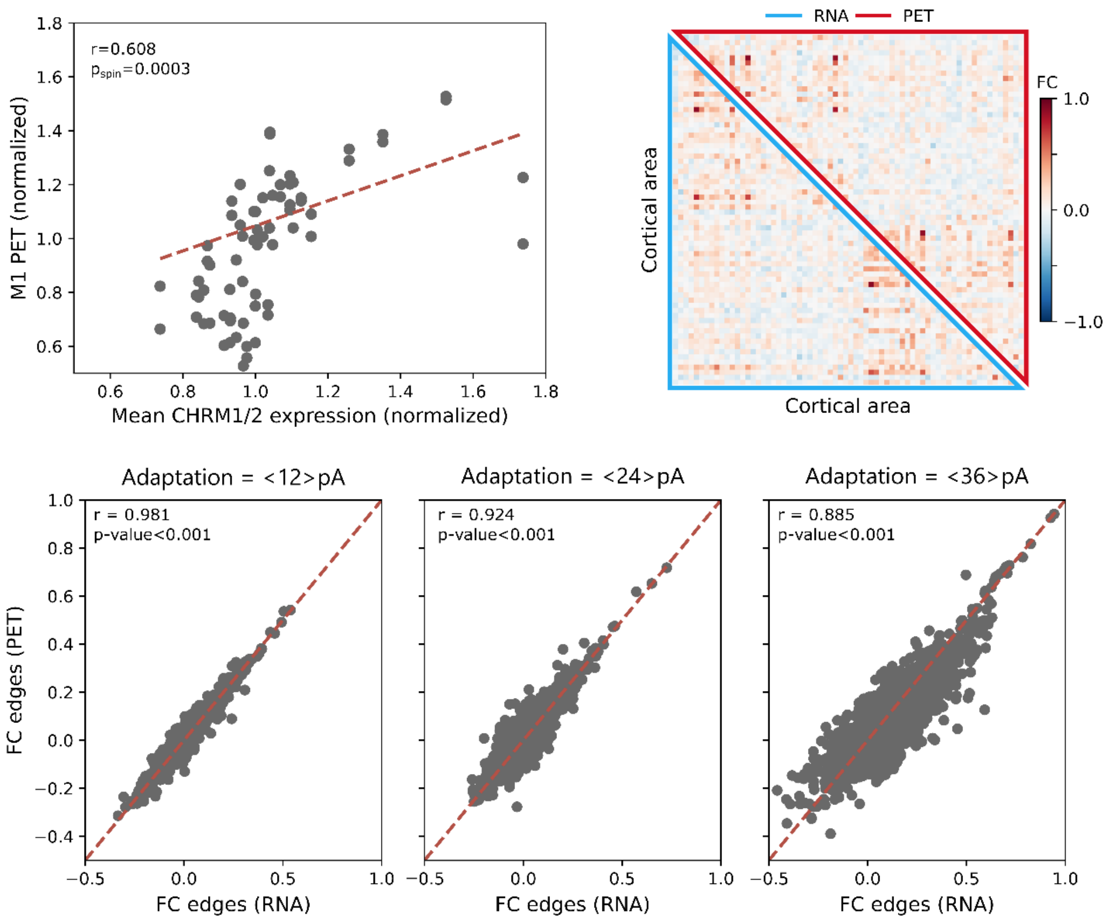

Fig. S3. Correspondence between transcriptomic and PET-derived muscarinic receptors maps. Top-left: Correlation between normalized positron emission tomography (PET) tracer affinity for muscarinic M1 receptors and normalized transcriptomic expression of muscarinic receptor subtypes M1 (CHRM1) and M2 (CHRM2) across cortical regions. PET-derived M1 binding was highly correlated with transcriptomic CHRM1/2 expression (Pearson's r=0.608, p_{spin}=0.0003). Dashed line indicates the fitted linear regression. Top-right: Simulated functional connectivity (FC; Pearson correlation) matrix at <b>=36pA. PET-derived FC is shown in the upper triangle (red), and transcriptomic-derived FC in the lower triangle (blue). Bottom-row: Edge-wise comparison of FC values derived from PET- and transcriptomic-constrained models across cortical networks for increasing levels of neuronal adaptation (b=<12>, <24>, and <36>pA). Each point represents a single connectivity edge between cortical regions (68 cortical areas, Desikan-Killiany parcellation). Dashed lines indicate identity (y = x). Pearson correlation coefficients and two-tailed p-values were used to assess statistical significance.

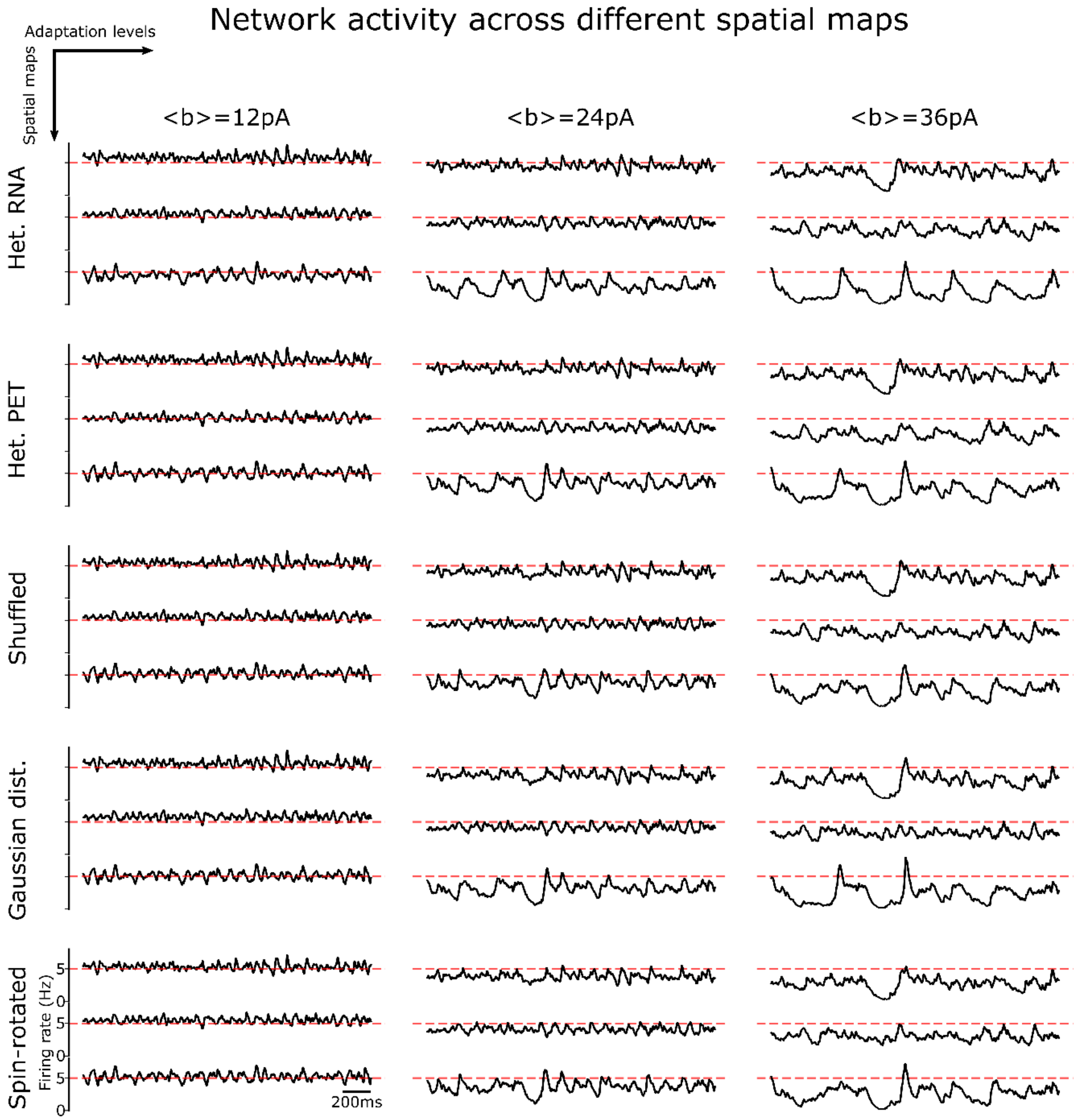

Fig. S4. Network dynamics across different spatial heterogeneity maps. Representative firing rate time series across awake-like (<b>=12pA), intermediate (<b>=24pA), and sleep-like (<b>=36pA) dynamical regimes for transcriptomic-constrained heterogeneous (Het. RNA), PET-constrained heterogeneous (Het. PET), shuffled, Gaussian-distributed, and spin-rotated spatial maps. Traces illustrate representative cortical regions with different adaptation levels (from higher to lower) within each condition. Red dashed lines indicate 5 Hz for reference.

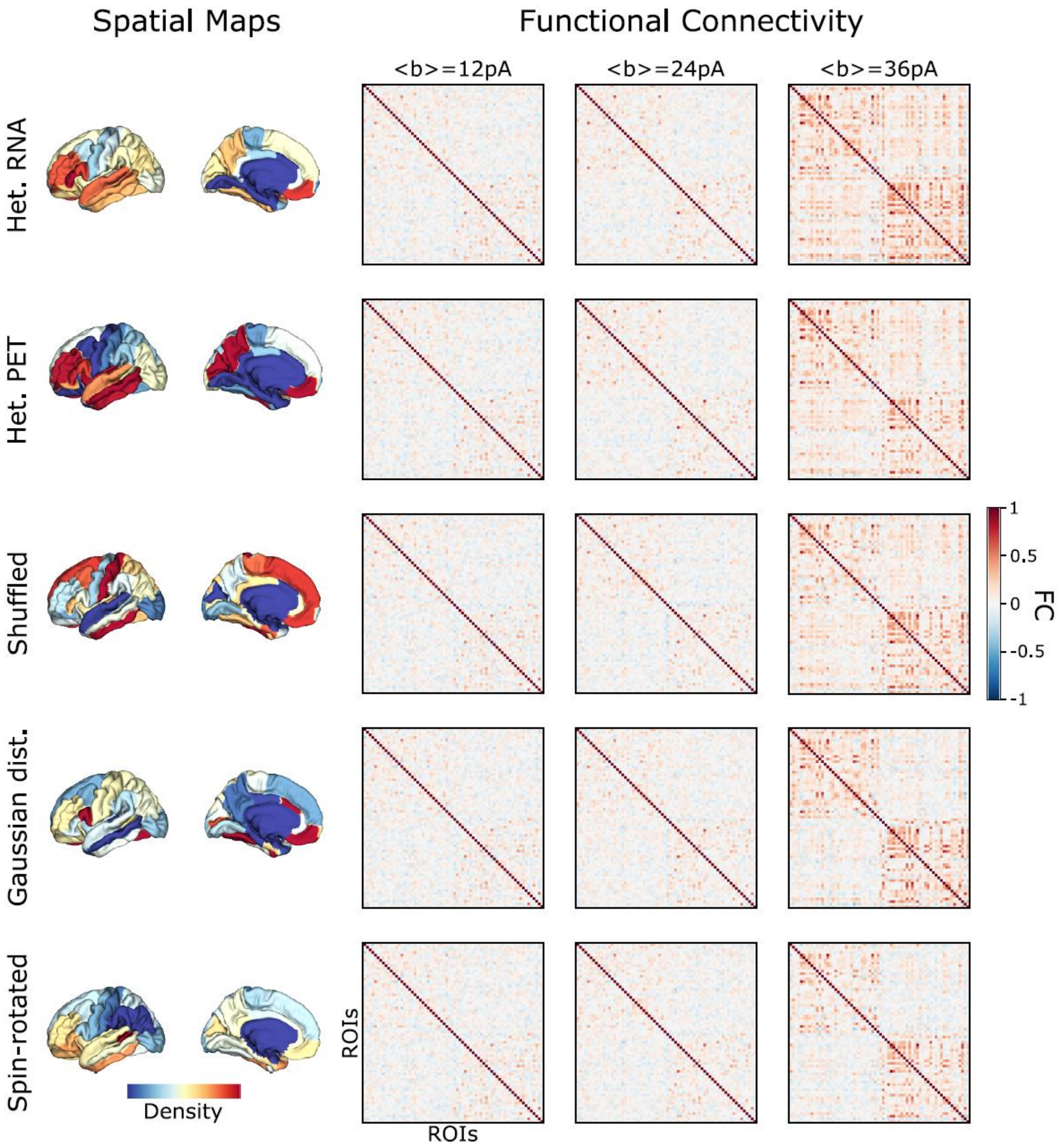

Fig. S5. Functional connectivity across different spatial heterogeneity maps. Spatial heterogeneity maps (left) and corresponding functional connectivity (FC; Pearson correlation) matrices (right) across awake-like (<b>=12pA), intermediate (<b>=24pA), and sleep-like (<b>=36pA) dynamical regimes for transcriptomic-constrained heterogeneous (Het. RNA), PET-constrained heterogeneous (Het. PET), shuffled, Gaussian-distributed, and spin-rotated spatial maps.

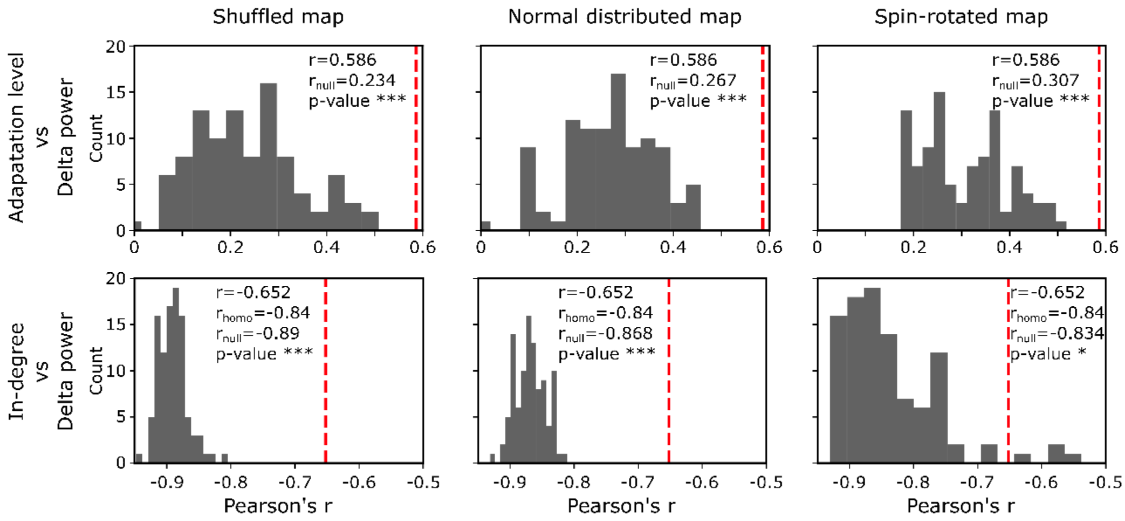

Fig. S6. Null model controls for relationships underlying localized sleep-like slow waves. Null distributions of Pearson correlation coefficients testing the relationships reported in Fig. 4 under different heterogeneity controls. Top row: correlation between regional adaptation level and delta power. Bottom row: correlation between in-degree connectivity and delta power. Columns show results obtained using spatially shuffled heterogeneity maps (left), random maps drawn from a Gaussian distribution matched for mean and variance (middle), and spin-rotated maps preserving spatial autocorrelation but disrupting anatomical alignment (right). Gray histograms represent correlations obtained from null models (100 runs; mean given by r_{null}), while red dashed lines indicate the correlations observed in the biologically aligned heterogeneous network (r). For reference, the correlation obtained in the homogeneous network (r_{homo}) is reported in the bottom panels. Permutation-based p-values were computed by comparing the observed correlation coefficient (red dashed line) to the null distribution of correlation coefficients obtained from surrogate heterogeneity maps.
